# Editorial: Detection and genomic evolution of viruses causing emerging and zoonotic infectious diseases

**DOI:** 10.3389/fcimb.2022.1009372

**Published:** 2022-09-21

**Authors:** Kai Huang, Esther Gan, Zengfeng Zhang

**Affiliations:** ^1^ One Health Laboratory, Department of Internal Medicine, University of Texas Medical Branch, Galveston, TX, United States; ^2^ Emerging Infectious Diseases, Duke-National University of Singapore (NUS) Medical School, Singapore, Singapore; ^3^ Department of Microbiology, Guangxi Medical University, Nanning, China

**Keywords:** infectious disease, zoonotic disease, evolution, genomics, RNA virus, emerging pathogen

## Introduction

Since the pandemic outbreak three years ago, COVID-19 has generated at least 12 existing variants and continues to evolve (National Center for Immunization and Respiratory Diseases (U.S.). Division of Viral Diseases, 2021; Tao et al., 2021). What drives this evolution is a mystery. There are other emerging or re-emerging zoonotic diseases, such as monkeypox or dengue, that pose a great threat to public health (Kozlov, 2022). The human-animal interface might be the hotspot facilitating these emergences, according to recent studies (The Lancet, 2020; Hedman et al., 2021). Zoonotic virus emergence is defined as being “naturally transmitted between people and vertebrate animals” in 3 classes: endemic, epidemic, and emerging and re-emerging (World Health Organization, 2014). To better understand the emergence, the evolution features of their genome, and the pathogen-host interactions, we proposed this Research Topic. We believe the key to preparing for the next pandemic is to closely and promptly monitor the emergence of such pathogens (and their variants) around the hotspot.

In this Research Topic, we invited global scholars to share their latest findings on the genomic signature of viruses that seriously threaten public health. We initially received 10 manuscripts but only 7 manuscripts were accepted for publication after rigorous peer reviews. Three of them are about the current most popular topic: the Severe Acute Respiratory Syndrome Coronavirus 2 (SARS-CoV-2), two of them regard the dengue virus (DENV), one is about the Chikungunya virus (CHIKV) and the last about the norovirus (NoV).

## SARS-CoV-2

SARS-CoV-2 has been the subject of heated discussion as the COVID-19 pandemic goes on. This topic contains one original research article, one opinion article, and one perspective article. In the original research article, Benslimane et al. depict the first COVID-19 wave in Qatar from 2020 to 2021. They successfully detect nine widely represented lineages with two Variants of Concern (VOCs). The B.1.428 was the dominant lineage before 2021, contributing 62% highest number worldwide but it was soon replaced by B.1.1.7 and B.1.351, possibly after multiple introductions from overseas. They decode the genome of SARS-CoV-2 and find some novel mutations that possibly occurred in Qatar.

The perspective article, based on the studies of Zahradnik et al., focuses on the evolution and mutations of the SARS-CoV-2 virus since early 2020. They identify 31 amino acid (AA) sites on spike protein (S-protein) under the convergent evolution after a careful exam. These 31 sites acquired 3 or more mutations independently in variable phylogenetic lineages, indicating they might be evolutionarily important with adaptive advantage regarding virus infectivity or spread. They find that almost all sites with high parallelism scores located in 3 critical structures: the N-terminal domain (NTD), receptor-binding domain (RBD), and the Furin cleavage site. More importantly, these AA sites nicely form co-localized patches on the above structures, revealing the functional involvements of these domains. They also observe that some mutations in the NTD domain co-dominate with certain RBD mutations whereas some mutations in NTD appear together but never alone. This suggests that synergic effects between mutations might exist.

SARS-CoV-2 host immune escape might be one of the important reasons that cause such a long pandemic. In the opinion article, Zhao et al. present one possible mechanism. SARS-CoV-2 Nsp1 is known to inhibit host antiviral interferon expression but does not affect the synthesis of viral proteins. Instead of simple inhibition or promotion of protein synthesis, they believe the Nsp1 modulates the ratio of viral/host mRNA translation but Nsp1 constrains the host more significantly than the virus. Nsp1 first binds to the SL1 of ribosome 40S, forming a structurally changed complex, which blocks its interaction with viral mRNA and then in turn causes the escape of viral mRNA from host RNase cleavage.

## Dengue virus

The two articles regarding the dengue virus include an original research article and a mini-review. In the original research, Hung et al. utilize deep sequencing and a machine learning algorithm to investigate 65 patients with primary infection or severe dengue. They find the frequency of single nucleotide variants (SNVs) and the defective virus genomes (DVGs) detection times were not only statistically different between those two groups but also positively correlated with disease severity. Thus, they develop an accurate model to estimate the risk of primary infection progressing into severe cases, useful for clinical prognosis and management. The mini-review by Bifani et al. summarizes the factors that influence DENV quasispecies diversity. They present an overview of interactions among the dengue virus quasispecies, the different mosquito vectors, the successive virus transmission, and the intra-host/inter-host barriers.

## CHIKV & NoV

Zou et al. evaluate chikungunya virus (CHIKV) infections occurring during the 2019 dengue epidemic in southwest China. Among the 697 tested patients, 693 (99.42%) were DENV-positive patients by sera RT-PCR test. The CHIKV co-infection rate was 12.33% (86 out of 697 patients). Full genomic sequences reveal that the four tested strains were Asian Genotype but lineage-specific mutations were present, potentially related to enhanced infectivity in mosquito vectors.

Xiong et al. apply nanopore metatranscriptomic sequencing to successfully identify the GII.12 [P16] norovirus (NoV), the first reported recombinant in China. The norovirus was responsible for a community-acquired acute gastroenteritis outbreak in 2020. The speed and ease of nanopore sequencing in the field would be beneficial for areas in which laboratory access is limited.

## Afterword

This Research Topic provides the most updated research advance from worldwide scientists regarding emerging zoonotic infectious diseases. We hope this Research Topic can be used to highlight the importance of continuous monitoring of emerging/re-emerging viruses.

## Author contributions

All authors contributed to co-editing the special Research Topic and edited, revised, and approved this editorial.

## Funding

This work was supported by the Centers for Excellence in Influenza Research and Surveillance (CEIRS) Program, Grant Number HHSN272201400008C, from the National Institute of Allergy and Infectious Diseases (NIAID).

## Acknowledgments

We sincerely appreciate all the authors submitting their manuscripts, every reviewer for the dedicated review effort, and the support from the journal.

## Conflict of interest

The authors declare that the research was conducted in the absence of any commercial or financial relationships that could be construed as a potential conflict of interest.

## Publisher’s note

All claims expressed in this article are solely those of the authors and do not necessarily represent those of their affiliated organizations, or those of the publisher, the editors and the reviewers. Any product that may be evaluated in this article, or claim that may be made by its manufacturer, is not guaranteed or endorsed by the publisher.

## References

[B1] Hedman H. D. Krawczyk E. Helmy Y. A. Zhang L. Varga C. (2021). Host diversity and potential transmission pathways of SARS-CoV-2 at the human-animal interface. Pathog. Basel Switz. 10, 180. doi: 10.3390/pathogens10020180 PMC7915269 33567598

[B2] Kozlov M. (2022). Monkeypox goes global: why scientists are on alert. Nature 606, 15–16. doi: 10.1038/d41586-022-01421-8 35595996

[B3] National Center for Immunization and Respiratory Diseases (U.S.). Division of Viral Diseases (2021) SARS-CoV-2 variant classifications and definitions. Available at: https://stacks.cdc.gov/view/cdc/105817 (Accessed July 21, 2022).

[B4] Tao K. Tzou P. L. Nouhin J. Gupta R. K. de Oliveira T. Kosakovsky Pond S. L.. (2021). The biological and clinical significance of emerging SARS-CoV-2 variants. Nat. Rev. Genet. 22, 757–773. doi: 10.1038/s41576-021-00408-x 34535792 PMC8447121

[B5] The Lancet (2020). Zoonoses: beyond the human-animal-environment interface. Lancet Lond. Engl. 396, 1. doi: 10.1016/S0140-6736(20)31486-0 32622381

[B6] World Health Organization (2014)Zoonotic disease: emerging public health threats in the region. Available at: http://www.emro.who.int/about-who/rc61/zoonotic-diseases.html (Accessed August15, 2022).

